# Elevated suPAR Is an Independent Risk Marker for Incident Kidney Disease in Acute Medical Patients

**DOI:** 10.3389/fcell.2020.00339

**Published:** 2020-06-12

**Authors:** Esben Iversen, Morten Baltzer Houlind, Thomas Kallemose, Line Jee Hartmann Rasmussen, Mads Hornum, Bo Feldt-Rasmussen, Salim S. Hayek, Ove Andersen, Jesper Eugen-Olsen

**Affiliations:** ^1^Department of Clinical Research, Copenhagen University Hospital Amager and Hvidovre, Hvidovre, Denmark; ^2^Icahn School of Medicine at Mount Sinai, New York, NY, United States; ^3^Department of Drug Design and Pharmacology, University of Copenhagen, Copenhagen, Denmark; ^4^Hospital Pharmacy, Copenhagen University Hospital Amager and Hvidovre, Hvidovre, Denmark; ^5^Department of Psychology and Neuroscience, Duke University, Durham, NC, United States; ^6^Department of Nephrology, Rigshospitalet, Copenhagen, Denmark; ^7^Department of Clinical Medicine, Medical Sciences, University of Copenhagen, Copenhagen, Denmark; ^8^Department of Medicine, Division of Cardiology, University of Michigan, Ann Arbor, MI, United States; ^9^Emergency Department, Copenhagen University Hospital Amager and Hvidovre, Hvidovre, Denmark

**Keywords:** chronic kidney disease, acute kidney injury, dialysis, soluble urokinase plasminogen activator receptor, glomerular filtration rate, emergency department

## Abstract

**Introduction:**

Identifying patients at high risk of developing kidney disease could lead to early clinical interventions that prevent or slow disease progression. Soluble urokinase plasminogen activator receptor (suPAR) is an inflammatory biomarker thought to be involved in the pathogenesis and development of kidney disease. We aimed to determine whether elevated plasma suPAR measured at hospital admission is associated with incident kidney disease in patients presenting to the emergency department.

**Materials and Methods:**

This was a retrospective registry-based cohort study performed at the Emergency Department of Copenhagen University Hospital Amager and Hvidovre, Hvidovre, Denmark. Patients were included in the study from November 2013 to March 2017 and followed until June 2017. Patients were excluded if they were diagnosed with kidney disease or died prior to index discharge. Plasma suPAR was measured at hospital admission, and the main outcome was time to incident kidney disease, defined by ICD-10 diagnosis codes for both chronic and acute kidney conditions. Association between suPAR and time to incident kidney disease was assessed by Cox proportional hazard regression analysis.

**Results:**

In total, 25,497 patients (median age 58.1 years; 52.5% female) were admitted to the emergency department and followed for development of kidney disease. In multivariable Cox regression analysis adjusting for age, sex, eGFR, CRP, cardiovascular disease, hypertension, and diabetes, each doubling in suPAR at hospital admission was associated with a hazard ratio of 1.57 (95% CI: 1.38–1.78, *P* < 0.001) for developing a chronic kidney condition and 2.51 (95% CI: 2.09–3.01, *P* < 0.001) for developing an acute kidney condition.

**Discussion:**

In a large cohort of acutely hospitalized medical patients, elevated suPAR was independently associated with incident chronic and acute kidney conditions. This highlights the potential for using suPAR in risk classification models to identify high-risk patients who could benefit from early clinical interventions. The main limitation of this study is its reliance on accurate reporting of ICD-10 codes for kidney disease.

## Introduction

Chronic kidney disease (CKD) is a major health issue affecting 8–16% of people worldwide (Jha et al., 2013). Patients with CKD have increased rates of cardiovascular disease, end-stage renal disease (ESRD), hospitalization, and death (Go et al., 2004; Astor et al., 2011; Gansevoort et al., 2013). Identifying patients at high risk of developing CKD may lead to earlier medical interventions that reduce morbidity and mortality associated with disease progression (James et al., 2010; Akbari et al., 2014). However, current measures of kidney function, such as estimated glomerular filtration rate (eGFR) and proteinuria, are insensitive predictors of incident kidney disease (Levey et al., 2009; James et al., 2010; Eloot et al., 2011).

Soluble urokinase plasminogen activator receptor (suPAR) is an endogenous signaling molecule and biomarker of chronic inflammation, and elevated suPAR levels have been associated with a range of diseases and mortality (Eugen-Olsen et al., 2010). In 2015, suPAR was described as a novel biomarker for eGFR decline and CKD in a cohort of cardiovascular patients (Hayek et al., 2015). Subsequent studies have replicated these results in healthy middle-aged participants (Schulz et al., 2017), patients with type 1 diabetes (Curovic et al., 2019), and children (Schaefer et al., 2017). Another study demonstrated that high suPAR in patients undergoing cardiac surgery was a risk factor for postoperative acute kidney injury (Mossanen et al., 2017). There is growing evidence that suPAR may reflect the pathogenesis of kidney disease, and it has been proposed that suPAR itself causes kidney disease by damaging renal podocytes (Wei et al., 2011; Hayek et al., 2017).

Patients admitted through the emergency department are characterized by high age and disease burden (Juul-Larsen et al., 2017), and they are at elevated baseline risk of developing kidney disease after discharge (Rohun et al., 2011). Routine suPAR measurements in such high-risk patient populations may help identify patients at highest risk of developing kidney disease and ultimately lead to clinical interventions that slow or prevent disease progression. Therefore, we tested the hypothesis that elevated suPAR measured at hospital admission is associated with the development of kidney disease.

## Materials and Methods

### Study Design and Setting

This was a retrospective observational cohort study performed at Copenhagen University Hospital Amager and Hvidovre. The initial patient population included all patients admitted to the Acute Medical Unit of the Emergency Department between November 18, 2013 and March 17, 2017 with follow-up data accessible in the Danish National Patient Registry (DNPR). Index admission was defined as the first recorded admission with a suPAR measurement within this study period. Patients were excluded from analysis if they were diagnosed with kidney disease, received dialysis, or died from any cause prior to index discharge. Patients with prior urolithiasis were not excluded. Data on prior kidney disease was obtained from the DNPR dating back to 1977. The study was approved by the Danish Data Protection Agency (ref. HVH-2014-018, 02767) and the Danish Health and Medicines Authority (ref. 3-3013-1061/1).

### Measurements

Demographic information including age and sex was obtained from the Civil Registration System. Chronic disease status including cardiovascular disease, hypertension, and diabetes was described by two-year history of chronic diseases based on International Classification of Disease (ICD)-10 diagnosis codes from the DNPR (Supplementary Table S1). Blood samples were collected on admission to the Acute Medical Unit, and a standard panel of markers, including creatinine, C-reactive protein (CRP), and suPAR, was analyzed as part of routine evaluation at the Department of Clinical Biochemistry. Creatinine was measured on a Roche Cobas^®^ c8000 701/702 (Roche Diagnostics, Mannheim, Germany) with a module instrument using the Roche Creatinine Plus version 2 IDMS-traceable enzymatic assay. CRP was measured on a Roche Cobas^®^ 6000 (Roche Diagnostics, Mannheim, Germany). suPAR was measured on a Siemens BEP2000 using the suPARnostic^®^ AUTO Flex ELISA (ViroGates A/S, Birkerød, Denmark) according to the manufacturer’s instructions. Estimated GFR was calculated with the creatinine-based equation from the Chronic Kidney Diseases Epidemiology Collaboration (CKD-EPI) (Inker et al., 2012).

### Outcomes

Information about disease status, hospital readmissions, and death was collected during a follow-up period beginning at index discharge and extending to a censor date (June 17, 2017) 90 days after the last patient was included (median follow-up time 755 days; range 90–1,307 days). Patients who left the country during the study period were censored at their last recorded hospitalization. Disease status during the study period was determined by ICD-10 codes obtained from the DNPR, which compiles information from all inpatient and outpatient activity within the Danish secondary healthcare system (Lynge et al., 2011). The quality of information within the DNPR has been described elsewhere (Schmidt et al., 2015). Kidney disease was defined as any ICD-10 diagnosis, procedure, or operation code suggesting renal impairment (Supplementary Table S2). Kidney diseases were categorized as *chronic kidney condition* (CKD, glomerular disease, tubulointerstitial disease, or other renal disorder) or *acute kidney condition* (acute dialysis or acute kidney injury). Information on vital status at the end of follow-up was obtained from the Civil Registration System. The primary outcome was time to event during follow-up, which was defined as number of days from index discharge to first kidney disease diagnosis or death (whichever came first).

### Statistics

Patient characteristics are presented with basic statistics: continuous variables as median with interquartile range, and discrete variables as number with percent of patients. Comparison between included and excluded patients was assessed by Wilcoxon rank sum test. Association between suPAR and incident kidney disease was assessed by Cox proportional hazard regression analysis and graphically represented by cumulative incidence plots. Association between suPAR and all-cause mortality was assessed by Cox proportional hazard regression analysis and graphically represented by a Kaplan-Meier plot.

An initial Cox regression model was generated with suPAR quartile as the explanatory variable and incident chronic kidney condition, acute kidney condition, or death as the endpoint. Start date was set to day of index discharge, censor date was set to June 17, 2017, and all endpoints were modeled as competing events for all other endpoints. Next, the analysis was repeated with suPAR modeled as a continuous variable. This was done first as a univariate model with log_2_(suPAR) as the sole explanatory variable and then as a multivariate model adjusted for age, sex, log_2_(eGFR), log_10_(CRP), cardiovascular disease, hypertension, and diabetes. In these models, suPAR, eGFR, and CRP were log-transformed for the purpose of interpretation and comparison with previous studies (Rasmussen et al., 2016; Rasmussen et al., 2018). Collinearity between log_2_(suPAR) and the covariates was assessed by variance inflation factor (VIF), and VIF greater than 5 was considered an indication of collinearity (Sheather, 2009). Association between log_2_(suPAR) and log_2_(eGFR) was further assessed by linear regression, and normality of residuals was evaluated by QQ-plot. Given the known association between suPAR and eGFR, an additional sensitivity analysis removing patients with eGFR less than 60 mL/min/1.73 m^2^ was performed for the multivariate models. For a similar reason, sensitivity analysis removing patients with prior diagnosis of cardiovascular disease was also performed for the multivariate models.

To investigate differences in hazard ratio for specific etiologies of chronic kidney conditions, the continuous Cox regression models were repeated for these endpoints subdivided by disease type (CKD, glomerular disease, tubulointerstitial disease, or other renal disorder). To investigate differences in hazard ratio for specific etiologies of acute kidney conditions, the continuous Cox regression models were repeated for these endpoints subdivided by specific time periods from index discharge to diagnosis (<30 days, 30–90 days, or >90 days). In these time period specific models, patients who were censored or developed an event before each time period were excluded from the model for that time period, and patients who were censored or developed an event after each time period were censored at the end of that time period. Proportional hazard assumption of each regression model was evaluated by Schoenfeld residuals. *P* < 0.05 was taken to be statistically significant. SAS Enterprise Guide 7.1 (SAS Institute Inc, Cary, NC, United States) was used for statistical analysis and generation of figures.

## Results

### Study Cohort

In total, 29,088 patients with follow-up data had suPAR measured at hospital admission during the study period. Of these, 2,836 patients were excluded due to prior kidney disease and 755 additional patients were excluded due to death from any cause prior to index discharge, resulting in a study cohort of 25,497 acute medical patients (Supplementary Figure S1). Baseline characteristics at index admission for the study cohort are shown in Table 1. Median age was 58.1 years, and 52.5% of patients were female. Compared with patients excluded due to prior kidney disease (n = 2,836), the study cohort had higher median eGFR (88.7 vs. 56.7 mL/min/1.73 m^2^), lower median CRP (4.0 vs. 16.0 mg/L), and lower median suPAR (2.7 vs. 4.5 ng/mL) (all *P* < 0.001). Compared with patients excluded for any reason (*n* = 3,591), the study cohort had higher median eGFR (88.7 vs. 57.4 mL/min/1.73 m^2^), lower median CRP (4.0 vs. 26.0 mg/L), and lower median suPAR (2.7 vs. 4.9 ng/mL) (all *P* < 0.001).

**TABLE 1:** Patient characteristics by outcome.

	All patients *N* = 25,497	Incident chronic kidney condition *n* = 570	Incident acute kidney condition *n* = 190	Death without kidney disease *n* = 3,199	No event (censor) *n* = 21,538
**Demographics**					
Age in years, median (IQR)	58.1 (40.3–73.7)	72.5 (58.2–81.8)	68.5 (56.3–77.0)	79.4 (69.2–87.2)	54.0 (37.4–69.6)
Female, *n* (%)	13,373 (52.5%)	265 (46.5%)	73 (38.4%)	1,697 (53.0%)	11,338 (52.6%)
**Chronic disease status**					
Hypertension, *n* (%)	2,745 (10.8%)	131 (23.0%)	38 (20.0%)	624 (19.5%)	1,952 (9.1%)
Cardiovascular disease, *n* (%)	2,534 (9.9%)	123 (21.6%)	35 (18.4%)	745 (23.3%)	1,631 (7.6%)
Chronic pulmonary disease, *n* (%)	2,160 (8.5%)	67 (11.8%)	36 (18.9%)	595 (18.6%)	1,462 (6.8%)
Diabetes, *n* (%)	1,601 (6.3%)	108 (18.9%)	44 (23.2%)	362 (11.3%)	1,087 (5.0%)
Cancer, *n* (%)	1,291 (5.1%)	42 (7.4%)	11 (5.8%)	653 (20.4%)	585 (2.7%)
Dementia, *n* (%)	505 (2.0%)	13 (2.3%)	3 (1.6%)	264 (8.3%)	225 (1.0%)
Liver disease, *n* (%)	362 (1.4%)	11 (1.9%)	11 (5.8%)	76 (2.4%)	264 (1.2%)
Peptic ulcer disease, *n* (%)	226 (0.9%)	11 (1.9%)	5 (2.6%)	54 (1.7%)	156 (0.7%)
Other^a^, *n* (%)	465 (1.8%)	19 (3.3%)	8 (4.2%)	100 (3.1%)	338 (1.6%)
**Hospital admission**					
Received surgery during hospitalization, *n* (%)	230 (0.9%)	3 (0.5%)	2 (1.1%)	24 (0.8%)	201 (0.9%)
Length of stay in days, median (IQR)	1 (0–3)	2 (1–6)	2 (1–7)	3 (1–8)	1 (0–2)
Acute readmission during follow-up:					
Within 30 days, *n* (%)	3,911 (15.3%)	154 (27.0%)	64 (33.7%)	1,119 (35.0%)	2,574 (12.0%)
Within 90 days, *n* (%)	6,058 (23.8%)	251 (44.0%)	101 (53.2%)	1,596 (49.9%)	4,110 (19.1%)
**Biomarkers**					
eGFR in mL/min/1.73 m^2^, median (IQR)^b^	88.7 (69.8–104.7)	54.0 (36.3–84.6)	72.6 (49.7–93.2)	69.8 (50.0–87.7)	91.5 (74.7–106.7)
<60 mL/min/1.73 m^2^, *n* (%)	3,987 (16.4%)	311 (57.2%)	68 (37.0%)	1,180 (38.2%)	2,428 (11.8%)
≥60 mL/min/1.73 m^2^, *n* (%)	20,323 (83.6%)	233 (42.8%)	116 (63.0%)	1,908 (61.8%)	18,066 (88.2%)
CRP in mg/L, median (IQR)^b^	4 (1–22)	10 (3–50)	9 (3–55)	19 (5–70)	4 (1–15)
suPAR in ng/mL, median (IQR)^b^	2.7 (2.0–3.9)	4.2 (2.9–5.8)	4.6 (3.1–6.3)	4.5 (3.3–6.2)	2.5 (1.9–3.5)

*^*a*^Includes connective tissue disease, paraplegia/hemiplegia, and HIV/AIDS. ^*b*^Measured at index admission; 1,187 patients (4.7%) missing value for eGFR and C-reactive protein. CRP, C-reactive protein; eGFR, estimated glomerular filtration rate; IQR, interquartile range; suPAR, soluble urokinase plasminogen activator receptor.*

### Outcomes During Follow-Up

During the follow-up period, 760 patients (3.0%) developed kidney disease, and 3,199 patients (12.5%) died without being diagnosed with kidney disease. Among patients who developed incident kidney disease, 570 patients (75%) developed a chronic kidney condition, and 190 patients (25%) developed an acute kidney condition (Table 1). Patients who developed kidney disease or died were older, had more comorbid conditions, more readmissions, lower eGFR, higher CRP, and higher suPAR at index admission compared with patients who were censored at follow-up. Basic statistics for patients developing each type of kidney condition are shown in Table 2. Among incident chronic kidney conditions, CKD was the most common diagnosis (263 cases), tubulointerstitial disease was the next most common (185 cases), and glomerular disease was the least common (23 cases). Among incident acute kidney conditions, 24 cases (12.6%) occurred within 30 days of index discharge, 29 cases (15.3%) occurred within 30–90 days, and 137 cases (72.1%) occurred after 90 days.

**TABLE 2:** Association of log_2_(suPAR) with development of incident chronic and acute kidney conditions.

Endpoint	No. patients	Median (IQR) eGFR^a^ (mL/min/1.73 m^2^)	Median (IQR)^a^ suPAR (ng/mL)	Hazard Ratio (95% CI)^b^	
				
				Univariate	Multivariate^c^
**Chronic kidney condition**	570	54.0 (36.3–84.6)	4.2 (2.9–5.8)	2.52 (2.31–2.75)	1.57 (1.38–1.78)
Chronic kidney disease	263	41.2 (30.6–58.4)	4.8 (3.6–6.3)	3.20 (2.84–3.60)	1.77 (1.47–2.13)
Glomerular disease	23	73.1 (52.8–98.0)	3.2 (2.0–5.8)	1.64 (1.00–2.67)	1.13 (0.60–2.14)
Tubulointerstitial disease	185	82.7 (56.8–107.0)	3.2 (2.2–4.6)	1.56 (1.31–1.86)	1.26 (1.00–1.59)
Other renal disorder	99	47.0 (33.6–71.5)	4.6 (3.5–6.3)	2.99 (2.46–3.65)	2.07 (1.56–2.76)
**Acute kidney condition**	190	72.6 (49.7–93.2)	4.6 (3.1–6.3)	2.96 (2.57–3.42)	2.51 (2.09–3.01)
<30 days from index	24	75.1 (59.3–93.5)	4.7 (3.9–6.3)	2.99 (2.01–4.44)	2.43 (1.45–4.06)
30–90 days from index	29	74.6 (54.0–93.6)	5.0 (3.1–7.4)	2.96 (2.06–4.26)	2.67 (1.70–4.20)
>90 days from index	137	69.9 (47.2–92.9)	4.6 (2.9–6.2)	2.96 (2.50–3.51)	2.49 (2.01–3.09)

*^*a*^Measured at index admission; 1,187 patients (4.7%) missing value for eGFR. ^*b*^Hazard ratios represent the proportional risk of developing the given kidney condition for each doubling in suPAR and is evaluated for the entire duration of follow-up unless otherwise specified. ^*c*^Adjusted for age, sex, log_2_(eGFR), log_10_(CRP), cardiovascular disease, hypertension, and diabetes. CI, confidence interval; eGFR, estimated glomerular filtration rate; IQR, interquartile range; suPAR, soluble urokinase plasminogen activator receptor.*

### Association of suPAR With eGFR

There was no indication of collinearity between log_2_(suPAR) and the covariates, with the largest variance inflation for age (VIF 1.82) and log_2_(eGFR) (VIF 1.74). However, there was a significant association between log_2_(suPAR) and log_2_(eGFR) at index admission, with β = –0.32 (Supplementary Figure S2). Patients with eGFR <60 mL/min/1.73 m^2^ had significantly higher suPAR compared to patients with eGFR >60 mL/min/1.73 m^2^ (median 4.5 vs 2.5 ng/mL, *P* < 0.001). Among patients with eGFR >60 mL/min/1.73 m^2^, 349 patients (1.7%) developed kidney disease, and 1,908 patients (9.4%) died during follow-up. Among patients with eGFR <60 mL/min/1.73 m^2^, 379 patients (9.5%) developed kidney disease, and 1,180 patients (29.6%) died during follow-up. For all categories of eGFR, patients who developed kidney disease or died during follow-up had significantly higher suPAR at index admission (Supplementary Figure S3).

### Association of suPAR With Chronic Kidney Conditions

**FIGURE 1:**
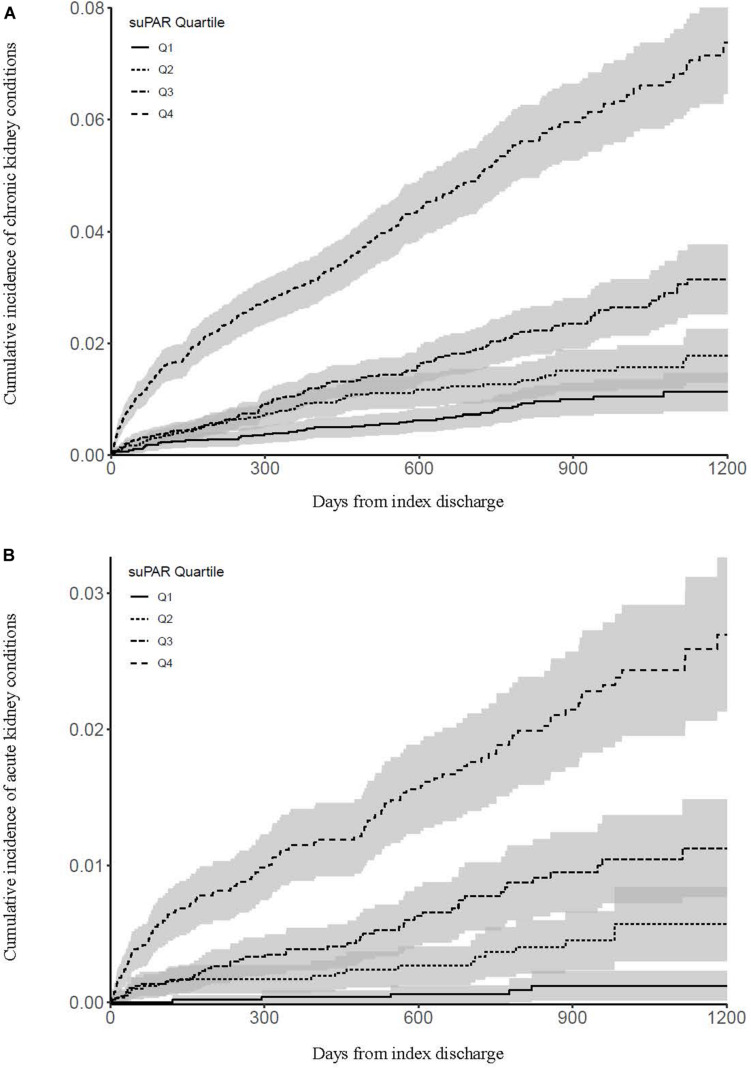
Cumulative incidence of chronic kidney conditions **(A)** and acute kidney conditions **(B)** by suPAR quartile. Shaded areas indicate 95% confidence intervals.

Cumulative incidence of chronic kidney conditions by suPAR quartile is shown in Figure 1A. Compared with patients in the lowest suPAR quartile, patients in the 2nd quartile had a hazard ratio of 1.65 (95% CI: 1.15–2.37, *P* = 0.007) for developing an incident chronic kidney condition, patients in the 3rd quartile had a hazard ratio of 2.77 (95% CI: 1.99–3.86, *P* < 0.001), and patients in the highest suPAR quartile had a hazard ratio of 8.53 (95% CI: 6.31–11.54, *P* < 0.001).

Modeling suPAR as a continuous variable, each doubling in suPAR yielded an unadjusted hazard ratio of 2.52 (95% CI: 2.31–2.75, *P* < 0.001) and an adjusted hazard ratio of 1.57 (95% CI: 1.38–1.78, *P* < 0.001) for developing an incident chronic kidney condition (Table 2). Sensitivity analysis removing patients with eGFR <60 mL/min/1.73 m^2^ resulted in an adjusted hazard ratio of 1.43 (95% CI: 1.17–1.73, *P* < 0.001), and sensitivity analysis removing patients with a prior diagnosis of cardiovascular disease resulted in an adjusted hazard ratio of 1.53 (95% CI: 1.33–1.77, *P* < 0.001) for developing an incident chronic kidney condition.

### Association of suPAR With Acute Kidney Conditions

Cumulative incidence of acute kidney conditions by suPAR quartile is shown in Figure 1B. Compared with patients in the lowest suPAR quartile, patients in the 2nd quartile had a hazard ratio of 4.83 (95% CI: 1.82–12.82, *P* = 0.002) for developing an incident acute kidney condition, patients in the 3rd quartile had a hazard ratio of 10.43 (95% CI: 4.14–26.23, *P* < 0.001), and patients in the highest suPAR quartile had a hazard ratio of 30.47 (95% CI: 12.44–74.67, *P* < 0.001).

Modeling suPAR as a continuous variable, each doubling in suPAR yielded an unadjusted hazard ratio of 2.96 (95% CI: 2.57–3.42, *P* < 0.001) and an adjusted hazard ratio of 2.51 (95% CI: 2.09–3.01, *P* < 0.001) for developing an incident acute kidney condition (Table 2). Sensitivity analysis removing patients with eGFR <60 mL/min/1.73 m^2^ resulted in an adjusted hazard ratio of 2.68 (95% CI: 2.17–3.32, *P* < 0.001), and sensitivity analysis removing patients with a prior diagnosis of cardiovascular disease resulted in an adjusted hazard ratio of 2.45 (95% CI: 2.01–3.00, *P* < 0.001) for developing an incident acute kidney condition.

### Association of suPAR With All-Cause Mortality

**FIGURE 2:**
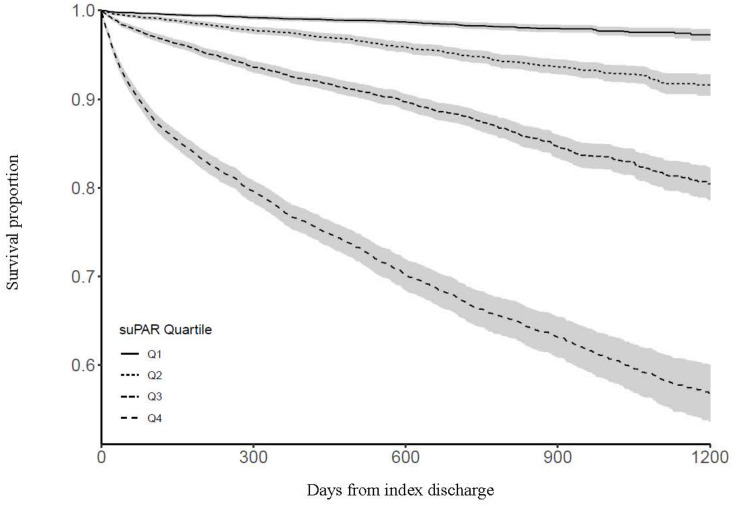
Survival by suPAR quartile. Shaded areas indicate 95% confidence intervals.

Survival by suPAR quartile is shown in Figure 2. Compared with patients in the lowest suPAR quartile, patients in the 2nd quartile had a hazard ratio of 3.17 (95% CI: 2.54–3.96, *P* < 0.001) for dying during follow-up, patients in the 3rd quartile had a hazard ratio of 7.99 (95% CI: 6.53–9.79, *P* < 0.001), and patients in the highest suPAR quartile had a hazard ratio of 23.23 (95% CI: 19.10–28.24, *P* < 0.001).

Modeling suPAR as a continuous variable, each doubling in suPAR yielded an unadjusted hazard ratio of 2.89 (95% CI: 2.79–3.00, *P* < 0.001) and an adjusted hazard ratio of 1.99 (95% CI: 1.89–2.09, *P* < 0.001) for dying during follow-up. Sensitivity analysis removing patients with eGFR <60 mL/min/1.73 m^2^ resulted in an adjusted hazard ratio of 2.02 (95% CI: 1.90–2.15, *P* < 0.001), and sensitivity analysis removing patients with a prior diagnosis of cardiovascular disease resulted in an adjusted hazard ratio of 2.01 (95% CI: 1.89–2.13, *P* < 0.001) for dying during follow-up.

## Discussion and Conclusion

In a cohort of 25,497 acutely hospitalized medical patients without prior kidney disease, we found that elevated suPAR at hospital admission was significantly associated with higher incidence of kidney disease and all-cause mortality during a median follow-up of 2 years. These associations were independent of age, sex, eGFR, CRP, cardiovascular disease, hypertension, and diabetes at index admission. In the adjusted model, each doubling in suPAR increased the hazard of developing an incident chronic kidney condition by 57% and the hazard of developing an incident acute kidney condition by 151%.

Our study cohort had notably lower median suPAR (2.7 ng/mL) than what has been reported by similar studies in the emergency department, such as the 2016 TRIAGE III trial in Denmark that reported median suPAR of 4.1 ng/mL (Schultz et al., 2018). This can be explained in part by our selection criteria. Patients with prior kidney disease or who died during index admission had higher median suPAR than the initial patient population, so their exclusion resulted in a study cohort with lower median suPAR (Supplementary Figure S1). Differences in the assays used to measure suPAR may also contribute to variations in suPAR values. A recent study comparing the assay used by our hospital (suPARnostic^®^ AUTO Flex ELISA) to another common assay (Quantikine^®^ Human uPAR-Immunoassay) found that suPARnostic^®^ yielded significantly lower suPAR in healthy controls and higher suPAR in patients with kidney disease (Winnicki et al., 2019).

While suPAR has previously been associated with kidney disease (Hayek et al., 2015; Mossanen et al., 2017; Schaefer et al., 2017; Schulz et al., 2017; Curovic et al., 2019), this is the first study to our knowledge to evaluate the association among patients in the emergency department. This is an important patient population to consider because these patients tend to be older and have multiple comorbid conditions (Juul-Larsen et al., 2017), putting them at greater risk of post-hospitalization kidney disease. Our results from a large population of acute medical patients suggest suPAR as a candidate to include in prediction models for kidney disease. If patients can be classified based on risk of developing kidney disease, then closer monitoring and early interventions in high risk patients may slow kidney disease progression and avoid complications related to renal dysfunction. For example, a 2009 study from Sweden found that one third of all adverse drug reactions leading to hospitalization in older patients were related to improper dose adjustment according to renal function (Helldén et al., 2009), emphasizing the importance of medication review for patients at risk of developing impaired renal function.

In 2013, our hospital began measuring suPAR as a routine biomarker in the emergency department. For acute medical patients, we have previously reported that elevated suPAR at hospital admission is associated with increased rates of surgery (Meyer et al., 2018), future readmission (Rasmussen et al., 2016), and all-cause mortality independent of presenting symptoms (Rasmussen et al., 2018). However, while suPAR has been implicated in many medical conditions, its causal mechanisms have only been investigated in kidney disease. It is believed that formation of a complex between suPAR and α_v_β_3_-integrin (a surface receptor on renal podocytes) can alter podocyte shape and disrupt the kidney’s ability to retain large proteins such as albumin (Hayek et al., 2017). We did not find that elevated suPAR was associated more strongly with kidney diseases affecting podocyte function, but this does not exclude the possibility that suPAR may directly contribute to kidney damage.

It may be suggested that suPAR is simply a sensitive filtration marker for early kidney damage, as suPAR is freely filtered by the kidneys and can be measured in the urine. However, we found that the association of suPAR with incident kidney disease remained significant even after adjustment for eGFR, suggesting that elevated suPAR also reflects a feature of kidney disease separate from decreased filtration. Another possibility is that suPAR is related to a systemic chronic inflammatory state. It is well established that chronic inflammation may be responsible for insidious tissue damage (Furman et al., 2019), and elevated suPAR is associated with multiple organ failure and sepsis (Patrani et al., 2016; Zeng et al., 2016). Therefore, high suPAR may indicate a state of chronic low-grade inflammation that predisposes a patient to kidney damage. We adjusted for CRP in our analysis, but CRP is a marker of acute inflammation and likely produced through an inflammatory pathway separate from that of suPAR (Lyngbæk et al., 2013).

In any case, our results in the context of current literature clearly indicate that suPAR is related to aspects of kidney function that are unexplained by current kidney biomarkers. If suPAR is involved in kidney disease pathogenesis, then interventions to reduce suPAR could potentially prevent disease progression in high-risk patients. Haupt et al. (2019) found that current lifestyle as well as changes to diet, smoking, alcohol use, and exercise impacted suPAR over a five-year period, and suPAR levels attained at the end of this period were predictive of follow-up mortality. This finding demonstrates that suPAR is a modifiable risk factor, but it is unknown whether initiatives to reduce suPAR in humans would also reduce the incidence of kidney disease. More recently, Hayek et al. (2020) observed that suPAR overexpression in mice led to acute kidney injury, and pretreatment with uPAR antibody attenuated this injury. This is the latest evidence to suggest a causative role of suPAR in kidney disease as well as identify a potential therapeutic target.

The strength of our study is its large population size. Copenhagen University Hospital Amager and Hvidovre was the first hospital to implement routine suPAR measurements for all acute medical patients, affording us a head start in investigating the role of systemic chronic inflammation in disease progression. Furthermore, since the DNPR contains information about all hospital encounters, we were able to obtain follow-up medical records for >99% of patients in our initial cohort. The collection of routine suPAR measurements and unique access to national registry data enabled us to investigate the association between suPAR and specific disease diagnoses over a four-year period in a large patient cohort.

The main limitation of our study is its reliance on ICD-10 diagnosis codes for both the exclusion criteria and primary outcome of kidney disease. However, a 2011 study found high positive predictive value of ICD-10 codes for renal disease within the DNPR (Thygesen et al., 2011), and a 2013 study concluded that registration of acute admissions within the DNPR has high validity (Vest-Hansen et al., 2013). Still, discrepancies between kidney disease prevalence and diagnosis within Denmark indicate that kidney disease is likely underreported in the DNPR (Henriksen et al., 2018). This discrepancy may be even greater for patients admitted through the emergency department, since clinicians sometimes only record the primary discharge diagnosis.

Ideally, our registry data would be supported by repeated measurements of both eGFR and proteinuria, as well as results of kidney biopsy. Current guidelines from Kidney Disease Improving Global Outcomes (KDIGO) define CKD as eGFR <60 mL/min/1.73 m^2^ or albuminuria >30 mg/day for a period of >3 months (Levin and Stevens, 2014). The lack of these endpoints within our cohort is an important limitation in the assessment of CKD, and our results rely largely on the accurate reporting of kidney disease in the DNPR. To address this issue, we performed a sensitivity analysis removing patients with eGFR <60 mL/min/1.73 m^2^ and obtained similar results. However, using a single creatinine measurement to identify kidney disease is inaccurate and does not always reflect a patient’s full clinical picture (Levey et al., 2015). For example, other conditions such as cardiovascular disease (Liu et al., 2014), hypertension (Lindeman et al., 1984), and diabetes (Alicic et al., 2017) are known to affect long-term kidney function. Therefore, we attempted to control for these conditions by including them in our analysis as potential confounders. We also performed a sensitivity analysis removing patients with known cardiovascular disease and obtained similar results.

In conclusion, we found that elevated suPAR in acute medical patients was independently associated with incident chronic and acute kidney conditions, as well as all-cause mortality. We propose suPAR as a candidate biomarker to include in risk classification models for kidney disease. Future studies are needed to evaluate whether early interventions in high risk patients can slow disease progression or avoid complications related to renal dysfunction.

## Data Availability Statement

The datasets generated for this study are available on request to the corresponding author.

## Author Contributions

Each author was involved in data analysis or contributed important intellectual content during manuscript writing, accepts personal accountability for their contributions, and agreed to ensure that questions pertaining to the accuracy or integrity of any portion of the work are appropriately investigated and resolved.

## Conflict of Interest

JE-O is a cofounder, shareholder, and Chief Scientific Officer of ViroGates A/S. JE-O and OA are named inventors on patents covering suPAR; the patents are owned by Copenhagen University Hospital Amager and Hvidovre, Hvidovre, Denmark and licensed to ViroGates A/S. LR has received funding for travel from ViroGates A/S. The remaining authors declare that the research was conducted in the absence of any commercial or financial relationships that could be construed as a potential conflict of interest.
