# Supplementary figures and images for: Elevated suPAR Is an Independent Risk Marker for Incident Kidney Disease in Acute Medical Patients
Source: Front Cell Dev Biol. 2020 Jun 12;8:339. doi: 10.3389/fcell.2020.00339 (PMC7303513; doi:10.3389/fcell.2020.00339)

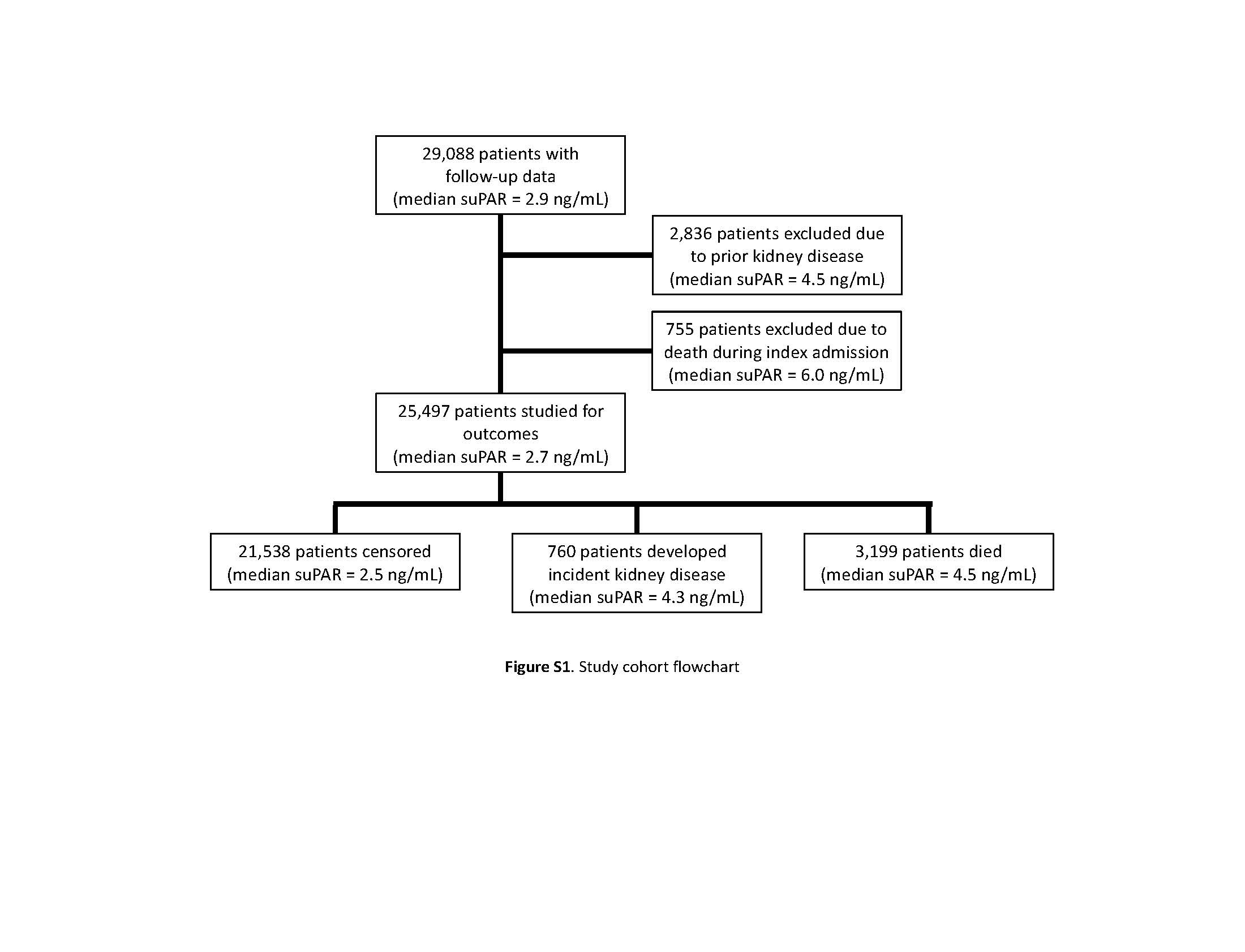

Supplement: Supplementary file 1 [file Image_1.JPEG]

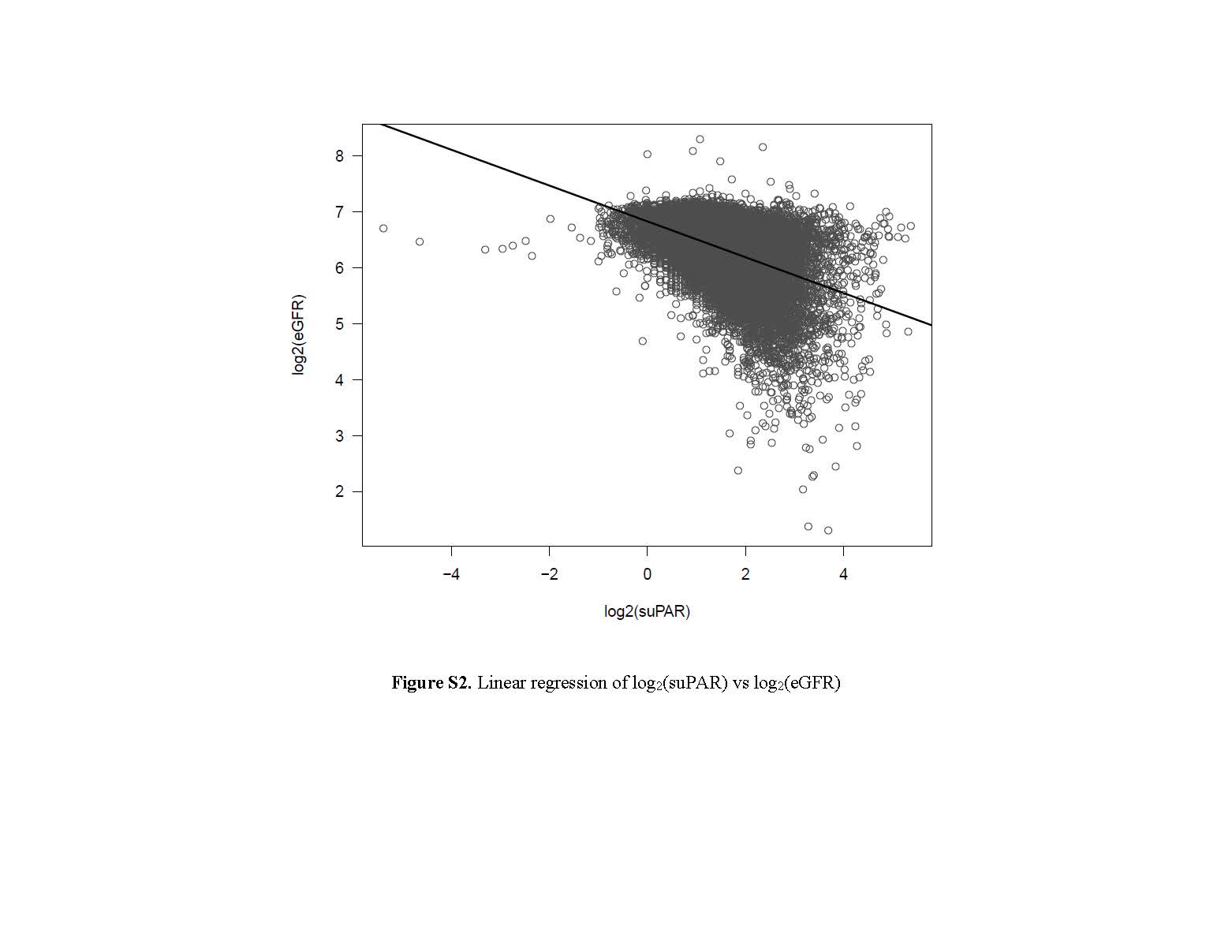

Supplement: Supplementary file 2 [file Image_2.JPEG]

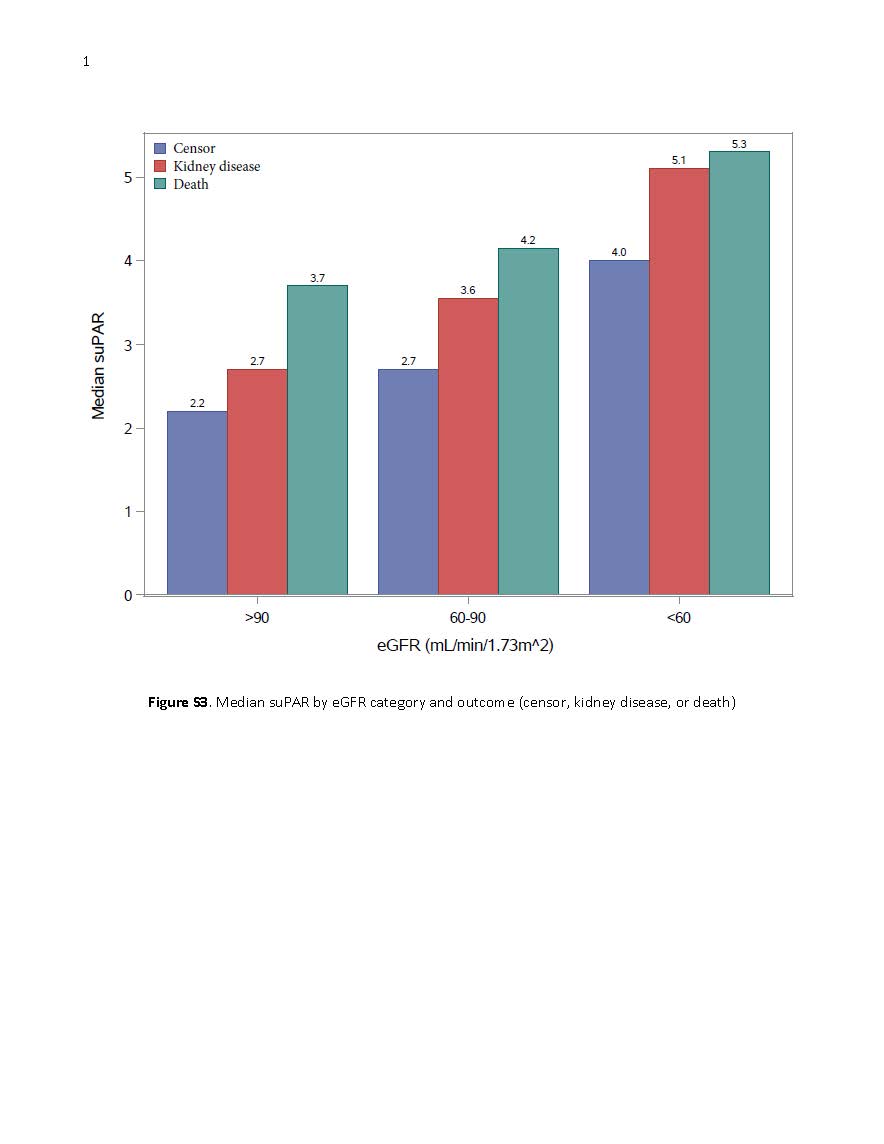

Supplement: Supplementary file 3 [file Image_3.JPEG]
