# Supplementary material for: Elevated suPAR Is an Independent Risk Marker for Incident Kidney Disease in Acute Medical Patients
Source: Front Cell Dev Biol. 2020 Jun 12;8:339. doi: 10.3389/fcell.2020.00339 (PMC7303513; doi:10.3389/fcell.2020.00339)
Supplement: Supplementary file 4 [file Table_1.DOCX]

| **Table S1.** ICD-10 codes for comorbid conditions | | | |
| --- | --- | --- | --- |
| **Condition** | **Category** | **ICD-10 codes** |  |
| **Cardiovascular disease** | Myocardial infarction | I21, I22, I25.2 |  |
|  | Congestive heart failure | I09.9, I110, I130, I132, I25.5, I42, I43, I50, P29.0 |  |
|  | Peripheral vascular disease | I70, I71, I73, I77.1, I79, K55, Z95 |  |
|  | Cerebrovascular disease | G45, G46, H34.0, I60, I61, I62, I63, I64, I65, I66, I67, I68, I69 |  |
| **Hypertension** |  | I10, I15 |  |
| **Diabetes** |  | E10, E11, E12, E13, E14 |  |
