# Supplementary material for: Elevated suPAR Is an Independent Risk Marker for Incident Kidney Disease in Acute Medical Patients
Source: Front Cell Dev Biol. 2020 Jun 12;8:339. doi: 10.3389/fcell.2020.00339 (PMC7303513; doi:10.3389/fcell.2020.00339)
Supplement: Supplementary file 5 [file Table_2.DOCX]

| **Table S2.** ICD-10 codes for kidney disease | | |  |
| --- | --- | --- | --- |
| **Condition** | **Category** | **ICD-10 codes** | |
| **Chronic kidney conditions^a,b^** | Chronic kidney disease | N18, N19, I12, I13, E08.22, E09.22, E10.22, E11.22, E13.22 | |
|  | Glomerular disease | N00, N01, N02, N03, N04, N05, N06, N07, N08 | |
|  | Tubulointerstitial disease | N10, N11, N12, N13, N14, N15, N16 | |
|  | Other renal disorder | N25, N26, N27, N28, N29 | |
| **Acute kidney conditions^c^** | Acute kidney injury | N17 | |
|  | Acute dialysis | BJFD0 | |
| ^a^Urolithiasis not included due to disease etiology  ^b^Chronic dialysis not included due to lack of incident cases  ^c^Kidney transplant not included due to lack of incident cases | | |  |
